# Chronological Changes in Neutrophil/lymphocyte Ratio in Advanced Gastric Cancer Patients Treated with Nivolumab: a Report of Nine Cases

**DOI:** 10.31557/APJCP.2020.21.10.2955

**Published:** 2020-10

**Authors:** Naohiko Nakamura, Shinichi Kinami, Jun Fujita, Daisuke Kaida, Yasuto Tomita, Takashi Miyata, Hideto Fujita, Hiroyuki Takamura, Nobuhiko Ueda, Takeo Kosaka

**Affiliations:** *Department of Surgical Oncology, Kanazawa Medical University Hospital, 1-1 Daigaku, Uchinada, Kahoku, Ishikawa, Japan. *

**Keywords:** Gastric cancer, nivolumab, neutrophil/lymphocyte ratio

## Abstract

**Background:**

Nivolumab has been approved for use in advanced gastric cancer (GC) after third-line chemotherapy in Japan. However, it remains difficult to predict favorable nivolumab response before treatment.

**Methods:**

We evaluated the clinical course with a focus on the chronological changes in neutrophil/lymphocyte ratio (NLR) throughout the chemotherapy and assessed the relationship between nivolumab response and chronological changes in NLR before nivolumab administration.

**Results:**

We experienced nine cases who received nivolumab monotherapy for unresectable advanced or postoperative recurrent GC. Nivolumab was used as third-line chemotherapy in all patients, and partial response (PR) and stable disease (SD) were observed in two patients each. Nivolumab treatment resulted in progressive disease (PD) in five patients. In patients with PR or SD, changes in the NLR tended to correspond to the response of target metastatic lymph nodes to first- and second-line chemotherapy. In the four cases with PR or SD following nivolumab, ∆NLR_responses_ that was the difference in the degree of decline during the most effective pretreatment chemotherapy were 1.39, 0.73, 1.62, and 1.22. However, the patients with PD showed lower ∆NLR_responses_, at 0.66, 0.66, 0.25, 0.13, and -0.05 in the five cases. Mean ∆NLR_responses_ in the patients with PR or SD and patients with PD were 1.17 and 0.33, respectively (P = 0.0008).

**Conclusions:**

We experienced nine GC cases treated with nivolumab and assessed the association between chronological NLR changes throughout chemotherapy and tumor response to nivolumab. Changes in NLR during pretreatment chemotherapy might predict tumor response to nivolumab monotherapy in patients with advanced GC.

## Introduction

Immune checkpoint inhibitors (ICIs) are now widely administered for the treatment of many kinds of cancer. The 2017 ATTRACTION-2 study showed that nivolumab monotherapy was more effective in patients with advanced gastric cancer (GC) (Kang et al., 2017). Although the response rate in nivolumab monotherapy was 11.2% in that study, it remains difficult to predict favorable nivolumab response before treatment. Recently, Ogata et al. reported that the neutrophil/lymphocyte ratio (NLR) may be an effective prognostic factor in patients with advanced GC receiving nivolumab treatment (Eisenhauer et al., 2009). In addition, there has been increasing interest in the association between the NLR and the clinical outcomes of upper gastrointestinal cancers. With regard to prognostic outcomes after gastrectomy in patients with GC, a retrospective analysis showed an association between a high NLR and poor survival (el-Hag and Clark, 1987). However, detailed chronological changes in the NLR have not been assessed in patients with advanced GC treated with multiple anticancer agents including nivolumab. An assessment of chronological changes in NLR throughout chemotherapy, especially during the nivolumab pretreatment period, might help to predict nivolumab monotherapy response in patients with advanced GC. We report our experience with nine patients with GC treated with nivolumab. We evaluated the clinical course with a focus on the chronological changes in NLR throughout the chemotherapy and assessed the relationship between nivolumab response and chronological changes in NLR before nivolumab administration. 

## Materials and Methods

We retrospectively analyzed the data of nine patients with unresectable advanced or postoperative recurrent GC who received nivolumab treatment at Kanazawa Medical University Hospital between September 2017 and December 2019. Data of clinical parameters such as sex, age, clinical course, and anticancer agents used for GC treatment were extracted from our hospital database. We calculated the NLR, defined as the ratio of neutrophil to lymphocyte counts in the white blood cell differential, from the results of blood examinations throughout the treatment. Using imaging studies, the patients were clinically staged based on the 7th edition of the American Joint Committee on Cancer (Ferlay et al., 2015) according to the depth of tumor invasion (T), extent of lymph node metastasis (N), and presence of distant metastasis (M). Responses of the target lesions to chemotherapy were classified according to the Response Evaluation Criteria in Solid Tumors (RECIST) guidelines (Kang et al., 2017). NLRresponse was defined as the NLR for the most effective response by RECIST obtained in first- or second-line chemotherapy regimens before nivolumab administration. NLRpre was defined as the NLR at initiation of the most effective regimen. ∆NLRresponse was defined as the difference in the degree of decline from NLRpre to NLRresponse. Data were expressed as mean (±standard deviation). Continuous variables were compared using Student’s t-tests. All statistical analyses were performed using JMP version 8.0 (SAS Institute, Cary, NC, USA).

We obtained informed consent from the patients. This study was approved by the Medicine Ethics Committee of Kanazawa Medical University.

## Results

*Patient characteristics and tumor response to nivolumab*

Nine patients in this study received nivolumab monotherapy; their characteristics are shown in Table 1. Mean patient age was 70.0 years, and 77.8% (n = 7) were men. Among the nine patients, four and five had postoperative recurrent and unresectable advanced GC, respectively. Seven patients underwent S-1-based chemotherapy as first-line chemotherapy, and seven patients underwent ramucirumab and/or (nab-) paclitaxel as second-line chemotherapy. Nivolumab was used as third-line chemotherapy in all nine patients. Following nivolumab monotherapy, partial response (PR) and stable disease (SD) were observed in two patients each, while five patients had progressive disease (PD).

*Chronological changes in NLRs and target lesions throughout chemotherapy*

**Figure 1:**
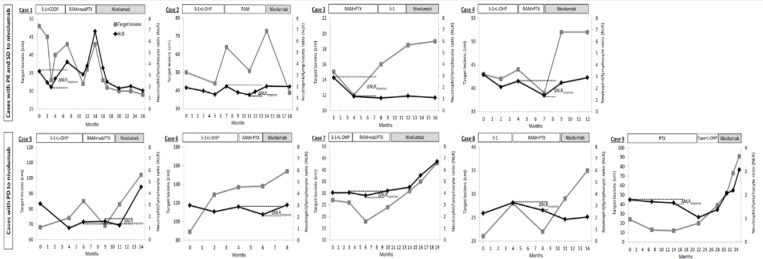
Gastric Cancer Treatments and Chronological Changes in NLR and Target Lesions in the Nine Cases. Case 1 and 2 showed partial response (PR) to nivolumab. Case 3 and 4 showed stable disease (SD) to nivolumab. Case 5, 6, 7, 8, and 9 showed progressive disease (PD) to nivolumab. Neutrophil/lymphocyte ratio, NLR; cisplatin, CDDP; ramucirumab, RAM; (nab-) paclitaxel, (nab-) PTX; oxaliplatin, L-OHP; capecitabine, Cape

In the two patients with PR following nivolumab treatment, the NLR decreased corresponding to the reduction in the diameters of the target metastatic lymph nodes in first- and second-line chemotherapy. However, the NLR at nivolumab initiation increased in both patients (Figure 1: Cases 1 and 2). The patients with SD following nivolumab treatment (Figure 1: Cases 3 and 4) showed decreased NLR corresponding to the regression of target metastatic lymph nodes following ramucirumab + paclitaxel administration. In contrast, the five patients with PD did not tend to show significant chronological changes in NLR corresponding to tumor response or significant decreases in NLR even if effective disease control was obtained following the first- or second-line chemotherapy (Figure 1: Cases 5–9).

*Correlation between ∆NLR*_response_ *and tumor response for nivolumab*

∆NLR_responses_ in the two cases with PR following nivolumab were 1.39 and 0.73, while that in the two cases with SD were 1.62 and 1.22. However, the patients with PD showed lower ∆NLR_responses_, at 0.66, 0.66, 0.25, 0.13, and -0.05 in the five cases (Table 2). Mean ∆NLR_responses_ in the patients with PR or SD and patients with PD were 1.17 and 0.33, respectively (P = 0.0008). The patients who achieved effective control with nivolumab therapy had a significantly higher ∆NLRresponse.

**Table 1:** Patient Characteristics and Chemotherapy for Gastric Cancer in the Nine Cases

Case	Gender	Age	Stage ^a)^	unresectabl/recurrence	1st line	2nd line	3rd line	Response for nivolumab
1	M	68	Stage IV	unresectable	S-1+Cisplatin	Ramucirumab+nab-Paclitaxel	Nivolumab	Partial response
2	F	71	Stage III	unresectable	S-1+Oxaliplatin	Ramucirumab	Nivolumab	Partial response
3	F	67	Stage III	recurrence	Ramucirumab+Paclitaxel	S-1	Nivolumab	Stable disease
4	M	70	Stage III	recurrence	S-1+Oxaliplatin	Ramucirumab+Paclitaxel	Nivolumab	Stable disease
5	M	66	Stage IV	unresectable	S-1+Oxaliplatin	Ramucirumab+nab-Paclitaxel	Nivolumab	Progressive disease
6	M	68	Stage IV	unresectable	S-1+Oxaliplatin	Ramucirumab+Paclitaxel	Nivolumab	Progressive disease
7	M	72	Stage IV	unresectable	S-1+Oxaliplatin	Ramucirumab+nab-Paclitaxel	Nivolumab	Progressive disease
8	M	71	Stage III	recurrence	S-1	Ramucirumab+Paclitaxel	Nivolumab	Progressive disease
9	M	77	Stage II	recurrence	Paclitaxel	Capecitabine+Oxaliplatin	Nivolumab	Progressive disease

^a)^ American Joint Committee on Cancer, 7^th^ edition [4]

**Table 2:** ∆NLR_response_ According to Tumor Response to Nivolumab in the Nine Cases

Case	Response for nivolumab	**∆**NLR_response_	Mean **∆**NLR_response_
1	PR	1.39	1.17**±**0.17
2	PR	0.73	
3	SD	1.62	
4	SD	1.22	
5	PD	-0.05	0.33**±**015
6	PD	0.66	
7	PD	0.25	
8	PD	0.66	
9	PD	0.13	

Mean ∆NLR_response_ ± standard deviation in patients with PR or SD (n = 4) and with PD (n = 5); Partial response, PR; stable disease, SD; progressive disease, PD

**Table 3:** Previous Case Reports in GC Patients Treated with Nivolumab

References	Age	Sex	Diagnosis	Chemotherapy prior to nivolumab	Response to nivolumab
Namikawa T, et al. 2018 [21]	77	M	GC with liver metastases after surgical resection	S-1+Oxaliplatin	CP
Togasaki K, et al. 2018 [22]	71	M	GC with liver metastases	Trastuzumab+Capeciabine+Cysplatin/Ramucirumab+Paclitaxel	PR
	79	M	GC with para-aortic lymph node metastasis	S-1+Cisplatin/Paclitaxel	PR
Satoyoshi R, et al. 2019 [23]	88	M	GC with liver metastases after surgical resection	None	PR
Kashima S, et al. 2019 [24]	25	M	GEC with metastatic lymph nodes after surgical resection	Trastuzumab+Capeciabine+Cysplatin	CR
Toyota S, et al. 2020 [25]	75	M	GC with peritoneal metastases	S-1+Oxaliplatin /Ramucirumab+Paclitaxel	PR
Tirino G, et al. 2020 [26]	78	F	GC with liver metastases	mFOLFOX-6 /Ramucirumab+Paclitaxel	CR
	82	M	GEC with liver metastases after surgical resection	mFOLFOX-6 /Ramucirumab+Paclitaxel	PR

Partial response, PR; stable disease, SD; progressive disease, PD; GEC, gastroesophageal cancer

## Discussion

We reported our experience with nine patients with GC receiving nivolumab monotherapy. We examined the chronological changes in NLR throughout chemotherapy and assessed the relationship between NLR changes during pretreatment chemotherapy and tumor responses to nivolumab monotherapy. 

GC is the fifth most common cancer and the third leading cause of cancer mortality worldwide (Kashima et al., 2019). Although some chemotherapy regimens have been shown to improve the survival outcomes of patients with advanced GC, the prognosis remains poor and hence further therapeutic development is needed. In this context, nivolumab, a fully human IgG4 monoclonal antibody against programmed death-1 (PD-1), has been approved for use in advanced GC after third-line chemotherapy in Japan and is expected to improve the prognosis of patients with advanced GC. However, it remains difficult to adjust the indication for nivolumab use and predict tumor response before nivolumab administration. Moreover, we are now facing challenges in choosing third-line chemotherapeutic agents between nivolumab or trifluridine/tipiracil (also known as TAS-102), a novel oral combination cytotoxic drug that has recently been reported to significantly improve overall survival in patients with advanced GC (Le et al., 2015). Thus, it is important to determine clinical parameters that can predict tumor response to nivolumab monotherapy.

The ATTRACTION-2 study reported a response rate of 11.2% and a median overall survival of 5.26 months (Edge and Compton, 2010). The present study retrospectively analyzed nine patients who received nivolumab monotherapy, two of whom (22.2%) showed PR and better prognosis. We excluded six patients in whom nivolumab monotherapy was initiated but was stopped after fewer than three administrations owing to the deterioration of their general condition because of GC progression. Because nivolumab is used as third- or later-line treatment for advanced GC and patients tend to show worse performance status (PS), the patients should show sufficient PS for the continuation of nivolumab treatment. All nine patients in this study had Eastern Cooperative Oncology Group performance status (ECOG-PS) scores of 0–1; thus, nivolumab could be continuously administered until imaging assessment of tumor response. Before nivolumab administration, eight patients were treated with S-1- or ramucirumab-based chemotherapy as first- or second-line therapies. These treatments reduced the target lesion diameter in four and seven patients, respectively. The tumor response based on the RECIST criteria in pretreatment chemotherapy was not associated with the response to nivolumab monotherapy. List of previous case reports in GC patients treated with nivolumab was shown Table 3. GC or gastroesophageal cancer patients underwent nivolumab monotherapy for liver metastases or lymph node metastases, and obtained good response to nivolumab. However, the chronological changes in NLR throughout chemotherapy were not assessed in these case reports.

This case report implied that ∆NLRresponse, defined as changes in NLR during pretreatment chemotherapy, might predict tumor response to nivolumab monotherapy in patients with advanced GC. Recent reports have indicated better prognosis in advanced GC patients with a low NLR before the first cycle of nivolumab or a reduction in NLR during nivolumab monotherapy (Eisenhauer et al., 2009; Nakamura et al., 2019). However, chronological changes in NLR during pretreatment chemotherapy have not been assessed as a predictor for tumor response in nivolumab monotherapy. Studies on ICIs have reported the association between their efficacy and PD-L1 or PD-L2 expression, mutation burden, and deficient mismatch repair (dMMR) (Namikawa et al., 2018; Ohmura et al., 2020). In addition, it was indicated that the expression levels of LAG-3 and OX40 on T cells correlated with the efficacy of nivolumab therapy (Ota et al., 2020). Compared to these parameters that could involve the antitumor mechanism of nivolumab, the NLR is a simpler index that could be calculated in routine examinations and used as a non-invasive biomarker. Elevated NLR is reportedly related to the poor prognosis of GC, colorectal, and lung cancer (Petrie et al., 1985; Shimada et al., 2010). We also previously showed an association between a high NLR and the presence of peritoneal metastasis in patients with advanced GC (Shimada et al., 2010). The mechanism underlying the association of ∆NLRresponse with tumor response to nivolumab remains unclear. Because the NLR can be considered an index reflecting systemic inflammatory response (Shitara et al., 2018; Tirino et al., 2020) and a decreased number of neutrophils promote lymphocyte activity (Tirino et al., 2010; Togasaki et al., 2018), cytotoxic or molecular targeted agents such as those administered during pretreatment chemotherapy for nivolumab could suppress systemic inflammation through tumor regression; the neutrophil count may result in a better response to nivolumab monotherapy due to increasing levels of activated lymphocytes.

This study has several limitations. This study has a retrospective design and a small sample size of only nine cases from a single institution. We report only an observed tendency regarding the relationship between changes in NLR and tumor response to nivolumab monotherapy. Thus, further retrospective or prospective cohort studies with sufficient sample sizes are needed to confirm the predictive value of the NLR for tumor response to nivolumab monotherapy.

In conclusion, we experienced nine cases administered nivolumab monotherapy for unresectable advanced or postoperative recurrent GC and assessed the association between chronological NLR changes throughout chemotherapy and tumor response to nivolumab. Changes in NLR during pretreatment chemotherapy might predict tumor response to nivolumab monotherapy in patients with advanced GC.

## List of abbreviations

Immune checkpoint inhibitors: ICIs; gastric cancer: GC; neutrophil/lymphocyte ratio: NLR; partial response: PR; stable disease: SD; progressive disease: PD; programmed death-1: PD-1; performance status: PS

## Declarations

Availability of data and materials: All data are available without restriction. Researchers can obtain data by contacting the corresponding author.
